# Strength of Ventral Tegmental Area Connections With Left Caudate Nucleus Is Related to Conflict Monitoring

**DOI:** 10.3389/fpsyg.2019.02869

**Published:** 2020-01-09

**Authors:** Ping C. Mamiya, Todd Richards, Neva M. Corrigan, Patricia K. Kuhl

**Affiliations:** ^1^Institute for Learning & Brain Sciences, University of Washington, Seattle, WA, United States; ^2^Department of Radiology, University of Washington, Seattle, WA, United States

**Keywords:** attention control, basal ganglia, midbrain, dopamine, executive function

## Abstract

Successful learning requires the control of attention to monitor performance and compare actual versus expected outcomes. Neural activity in the ventral tegmental area (VTA) has been linked to attention control in animals. However, it is unknown whether the strength of VTA connections is related to conflict monitoring in humans. To study the relationship between VTA connections and conflict monitoring, we acquired diffusion tensor imaging (DTI) data on 50 second language learners who we have previously studied. We performed probabilistic tractography to document VTA connections with the dorsal striatum and the anterior cingulate cortex (ACC), and administered the Flanker task in which subjects were required to monitor and report conflicts in visual stimuli. Reaction times (RTs) indexed students’ conflict monitoring. Probabilistic tractography revealed distinct neural connections between the VTA and the dorsal striatum and ACC. Correlational analyses between tractography and flanker RTs revealed that the strength of VTA connections with the left caudate nucleus was negatively correlated with RTs recorded in the presence of conflicts. This provides the first evidence to suggest that VTA connections with the left caudate nucleus are related to conflict monitoring in humans.

## Introduction

The cognitive ability to monitor potential conflicts and adjust behavioral performances is crucial for adaptive behavior. It enables individuals to direct attention, monitor performance and compare actual versus expected outcomes. In bilingual individuals, conflict monitoring enables people to detect differences in their two languages and switch from one language to the other. Thus, conflict monitoring helps avoid language conflicts. It has been shown that bilingual children and adults perform better in conflict monitoring tasks than matched monolingual controls, and this has been attributed to their experience in monitoring differences in different language systems (Bialystok et al., 2005).

There is evidence that dopamine is important for learning and monitoring conflicts in tasks (for a review, see Schultz, 2016). A commonly used conflict monitoring task, the Flanker task, has been used to assess the effects of dopamine on monitoring and reporting conflicts in human volunteers (i.e., Bunge et al., 2002a; Costa et al., 2008; Treit et al., 2014; Ong et al., 2017). Studies using either dopamine antagonists or dopamine receptor binding assays have shown that dopamine release is related to Flanker responses. Badgaiyan and Wack (2011) used positron emission tomography scans to demonstrate that there was a significant increase in dopamine release in the left caudate nucleus when subjects faced conflicting arrows in the Flanker task (Badgaiyan and Wack, 2011). Zirnheld et al. (2004) administered a non-selective dopamine receptor blocker, haloperidol, to healthy volunteers and found that subjects made more errors in detecting and reporting a targeted image 3 h after the treatment (Zirnheld et al., 2004). Another study by Rabella et al. (2016) using a different non-selective dopamine receptor blocker, Risperidone, also demonstrated a similar behavioral effect on the Flanker task in healthy volunteers (Rabella et al., 2016). Subjects showed increased reaction times (RTs) to detect and report a targeted image after receiving a single-dose of Risperidone.

Conversely, other studies administered a dopamine precursor, Levodopa, and found that increasing brain dopamine levels facilitated task performance. Healthy volunteers recognized new words written in a new language that they had just learned more accurately after they received Levodopa treatments (Knecht et al., 2004; Shellshear et al., 2015).

Aside from human studies, electrophysiological recordings from midbrain dopaminergic neurons in monkeys also found that dopaminergic neurons in the ventral tegmental area (VTA) were excited when animals were finding a correct target among distractors (Matsumoto and Takada, 2013). The release of dopamine in the left caudate nucleus in humans and the excitation of dopaminergic neurons in the VTA in monkeys highlight the importance of VTA-caudate nucleus connectivity during conflict monitoring. Although conflict monitoring can be impeded by reducing dopamine signaling through systematic administration of non-selective dopamine receptor blockers, it remains unclear whether the strength of anatomical connections between the VTA and the left caudate nucleus is directly linked to subjects’ performance in the Flanker task.

To study this question, we examined the brain connectivity between the VTA and the left caudate nucleus. We also examined the brain connectivity between the VTA and the anterior cingulate cortex (ACC). The ACC showed activations during the Flanker task in humans (Botvinick et al., 1999; Casey et al., 2000; Hazeltine et al., 2000; Van Veen et al., 2001; Bunge et al., 2002b; Luk et al., 2010; Abutalebi et al., 2012). We used diffusion tensor imaging (DTI) and characterized the locations and trajectories of the VTA connections to these brain regions. To index the strength of brain connectivity, we computed fractional anisotropy (FA) from DTI. FA in cortico-cortical or subcortico-cortical brain connections is related to a variety of cognitive functions and learning skills in children and adults (Klingberg et al., 2000; Tuch et al., 2005; Keller and Just, 2009; Forstmann et al., 2010, 2012; Yeatman et al., 2012; Qi et al., 2015; Mamiya et al., 2016, 2018). It is heavily influenced by the structural properties of brain connections, including the coherence of neural pathways, the organization of fiber crossing in a local environment and the amount of myelination (Douaud et al., 2011). It has been shown that better structural properties of brain connections are associated with higher FA. Individuals with higher FA exhibited improved reading skills (Keller and Just, 2009; Yeatman et al., 2012), better second language learning outcomes (Qi et al., 2015; Mamiya et al., 2016) and higher inhibitory control skills (Mamiya et al., 2018). Using FA, we wanted to examine whether the strength of the VTA connections to the left caudate nucleus or to the ACC was related to subjects’ performance in the Flanker task. We hypothesized that individuals with higher FA would show better conflict monitoring in the Flanker task.

## Materials and Methods

### Selection Criteria

All subjects were full-time students newly enrolled at the University of Washington (female = 24, male = 26). All experimental procedures were approved by the Institute Review Board of the University of Washington, and written informed consent was obtained from each student. The mean age of participants was 20.34 years of age (SEM = 0.44). All students acquired English in schools as a part of the curriculum and were part of the cohort that has been previously reported (Mamiya et al., 2016). The mean age of learning English words was 8.4 years old (SEM = 0.32). To ensure that all students had equivalent levels of English proficiency at the time of the study, we assessed their scores from the Test of English as a Foreign Language (TOEFL) test. All students showed similar proficiency levels in their reading, speaking, writing and listening tests [For reading: 26.87 (SEM = 0.38); for speaking: 22.06 (SEM = 0.29); for writing: 24.94 (SEM = 0.33); for listening: 25.84 (SEM = 0.42)]. Exclusion criteria were: previous residence outside of China prior to moving to the United States; previous participation in a student exchange program outside of China; both parents not of Chinese origin; past use of serotonin or dopamine related agents; a medical history of Axis I disorders, epilepsy, or brain injury; problems with normal vision or hearing; left-handedness as assessed by the Edinburgh handedness test. For MRI safety, individuals with metallic or cardiac implants or tattoos were additionally excluded.

### Flanker Task

We used the Flanker task to assess attention control. The experiment was conducted using a MacBook Pro with a Retina 15-inch display and a screen resolution of 2880-by-1800 at 220 pixels per inch. A MATLAB script was used to display five arrows on the laptop screen. There were two conditions in the task, congruent versus incongruent. We used a single block design and intermixed the congruent and incongruent trials within the block. Students were required to identify whether the arrow in the center of the image pointed in the same direction (congruent condition) or in the opposite direction (incongruent condition) of the flanking arrows. All students were instructed to press either the left-arrow or the right-arrow key on the keyboard to respond. The left-arrow key would be pressed if the center image pointed to the left, and the right-arrow key would be pressed if the center image pointed to the right. All students were instructed to respond as quickly as possible. The laptop recorded the time each student took to press the key after the presentation of arrows on the monitor in every trial. An invalid trial was marked if there was no key press after the presentation of five arrows for 2 s. All students had to make 100 correct trials in order to finish the task, and all of them successfully completed the task. We averaged students’ RT recorded in the congruent and the incongruent trials separately. Only trials with correct responses were correlated with FA values in brain voxels derived from our probabilistic tractography analysis.

### Brain Image Acquisition

Diffusion tensor imaging data were acquired on a Philips 3T Achieva scanner (v3.26) using an eight-channel head coil. An echo-planar diffusion spin-echo pulse sequence was used with the following parameters: 64 diffusion gradient directions, *b* value = 1,500 mm^–2^, TR = 8.986 ms, TE = 77 ms, acquisition matrix size 136 × 133 × 76, acquisition voxel size: 1.76 mm × 1.8 mm × 1.8 mm, reconstructed voxel size: 1.5 mm × 1.5 mm × 1.8 mm, EPS factor 47, receiver bandwidth 2,160 Hz, sound pressure 18.46 dB, fold-over direction AP, fat shift direction posterior (P) for TOPUP and anterior (A) for TOPDOWN, slice thickness = 1.8, SENSE factor 3 in the anterior-posterior direction, scan duration 24 min and 24 s for both TOPUP and TOPDOWN. To correct for the susceptibility-induced off-resonance field, DTI data was collected with reversed phase-encode blips, resulting in pairs of images with distortions going in opposite directions, followed by a method that was previously described and implemented in the FSL (Andersson et al., 2003; Smith et al., 2004). The two images were combined into a single corrected one.

To ensure the quality of DTI image, we adopted the procedures published by Liu et al. (2010) in addition to our careful visual inspections, to assess motion artifacts in all students’ DTI images. The method derived from the Liu et al. (2010) assessed the quality of DTI images by using the normalized correlation (NC) between successive slices across all the diffusion gradients. Thus, it enabled us to detect problems may have resulted from motions, or technical problems occurred during image acquisitions associated with magnetic field gradients or radio frequency. The NC computed pixel-wide cross correlation and normalized it by the square root of the autocorrelation of images:

NC⁢(A,B)=∑(Ai⋅Bi)i=1N∑Aji=1N⋅∑Bji=1N

A*_*j*_* was the *j*-th pixel of image A and B*_*j*_* was the *j*-th pixel of image B. *N* is the number of pixels that were considered in the image.

Based on our DTI image acquisition protocol, each student had 4,800 slices. We set the threshold of 3.0 as recommended. Single slices with indexes above 3.0 would be discarded due to the presence of motion artifact. Among all 4,800 slices examined, three subjects had 19 slices (0.4%) with motion artifact, 22 subjects had 11–18 slices with motion artifact (0.3%) while the remaining students showed less than 10 slices with motion artifact (0.2%) (Supplementary Figure S1). Based on the results of this analysis, all slices of students’ DTI images were used for probabilistic tractography analysis.

#### Seed and Target Masks

We set a seed mask to encompass the VTA. The VTA mask was created using the following three steps: we first used the MNI coordinates published by Harley et al. in the MNI T1-152 standard space (Hadley et al., 2014), and created a sphere encompassing 57 voxels that conforms the size of the VTA in human brains (Mai et al., 2015). Secondly, we transformed the VTA mask from the MNI to a subject’s DTI space. This was achieved by using the FSL command, flirt. This calculation allowed us to convert the mask between MNI and DTI spaces. Finally, we visually inspected the output to ensure the accuracy of transformation of the VTA mask to each subject’s DTI image.

We used the Juelich Histological Atlas provided by the FSL and selected the caudate nucleus as a region of interest (ROIs) in the MNI152 T1-1 mm brain. The ROI was used as a target mask in individual probabilistic tractography. For the ACC, we included the cingulum bundle when creating masks to ensure robust fiber tracking. We manually drew ACC on the MNI152 T1 1 mm brain using anatomical landmarks (Paus, 2001). After the target masks were created, we transformed the masks from the MNI to diffusion space using the FSL command, flirt.

#### Termination Masks

Termination masks were applied to restrict fiber tracking to the ACC. We applied the termination mask that covered brain areas between the bilateral ACC. The termination masks were transformed to each subject’s diffusion space.

### Tractography Analysis

We used the FMRIB’s diffusion toolbox (FDT) in FSL 5.0.5 Diffusion Toolbox^1^ (FDT) and carried out a two-step probabilistic tractography analysis (Behrens et al., 2003, 2007). This included (1) using Bayesian Estimation of Diffusion Parameters Obtained using Sampling Techniques (BEDPOSTX) available in FDT to estimate crossing fibers in addition to the principal diffusion directions estimation, and (2) performing Probabilistic Tracking with Crossing Fibers (probtrackx software) to compute the connectivity map using the number of samples starting from a seed mask and ending at a target mask, with the maximal number of 5,000. We limited curvature of angle from one voxel to the proceeding voxel to no greater than 80 degrees. Tracts generated from probabilistic tractography were used to isolate brain voxels in each individual’s FA map. Mean FA values were calculated in respective neural pathways in each individual. Respective neural pathways were binarized and then multiplied to show the overlap across subjects. Overlap maps were averaged to produce a group-level map, thresholded at 0.5 (to ensure that at least 5 out of 10 subjects showed a continuous tract between the VTA and a targeted brain region.

### Statistical Analysis

Partial correlation analysis in the Statistical Toolbox in the MATLAB was used for assessing relationships between FA values and students’ ΔRTs in the Flanker task while taking into the account of the effects of age on FA. Pearson’s correlation analysis was used to determine the relationship between students’ RTs recorded in the congruent/incongruent conditions and the effect of interference (ΔRTs). A two-tailed one-sample *t*-test was used to assess the differences between students’ RTs recorded in the congruent versus incongruent condition. Non-parametric Kruskal–Wallis tests were used to assess the differences in the AoA, TOEFL scores, RTs and accuracy between students with a negative versus positive ΔRT. Bonferroni correction was subsequently applied for the statistical results to account for the number of TOEFL scores and the number of conditions in the Flanker task. It was also applied for the number of brain regions in the Flanker-FA association analyses. A *p* level of 0.05 was used for statistical significance.

## Results

### Reaction Times (RTs) Recorded in the Incongruent Condition Were Significantly Correlated With the Effect of Interference (ΔRTs)

**FIGURE 1:**
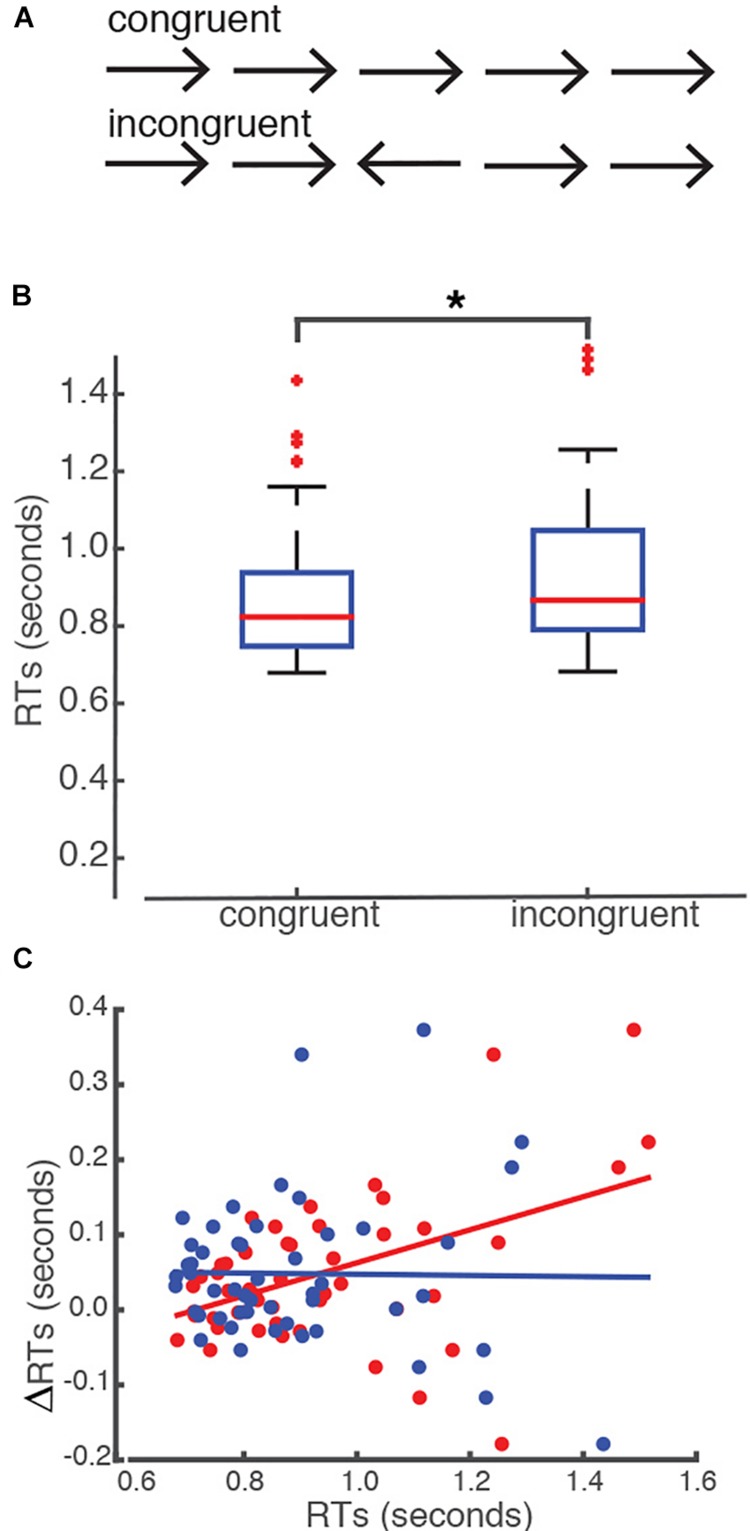
Flanker task. **(A)** Five arrows were presented in a single trial. There were two conditions of presentation: congruent versus incongruent. **(B)** Subjects took a significantly longer time to respond to the incongruent condition than to the congruent condition. The asterisk indicates a significant level of *p* = 0.05. The upper level of the box represents the 75th percentile whereas the lower level of the box represents the 25th percentile of subjects’ reaction times in each condition. The red lines represent the median reaction time for each condition. **(C)** The effect of interference (ΔRTs) was significantly correlated with the reaction times (RTs) recorded in the incongruent condition, but not with the ones recorded in the congruent condition. Red dots represent RTs recorded in the incongruent condition. Blue dots represent the RTs recorded in the congruent condition. RTs recorded in the incongruent condition significantly correlated with the ΔRTs, where the RTs recorded in the congruent condition did not (*p* > 0.05). *X*-axis represents the RTs and the *Y*-axis represents the differences in RTs between the incongruent and congruent conditions (ΔRTs).

We assessed subjects’ responses in two different conditions: congruent versus incongruent (see section “Materials and Methods” for details) (Figure 1). Our behavioral analysis revealed that all students showed equivalent rates of accuracy of response in both conditions. The average number of correct trials that the students made was 50.16 (SEM = 0.4) and the average rate of accuracy was 97.4% (SEM = 0.0043) in the congruent condition. In the incongruent condition, the average number of correct trials that subjects made was 49.84 (SEM = 0.4) and the average rate of accuracy was 97.7% (SEM = 0.0036). We did not find a statistical difference between congruent and incongruent conditions in terms of the number of correct trials, *t*(49) = 0.3937, *p* = 0.6955, or the rate of accuracy, *t*(49) = −0.7169, *p* = 0.48. However, we found that students required a significantly longer time to press the key in the incongruent condition as compared to the congruent condition. The mean RT recorded in the incongruent condition was 0.93 s (SEM = 0.0293) as opposed to 0.89 s (SEM = 0.026) in the congruent condition *t*(49) = –3.4178, *p* = 0.0013 (Figure 1B). Importantly, we found that the difference in RTs recorded between the congruent and incongruent conditions (ΔRTs) was significantly correlated with the RTs recorded in the incongruent condition (Pearson’s *r* = 0.412, *p* = 0.0014, corrected for multiple comparisons, Figure 1C), but not with the RTs recorded in the congruent condition (*p* > 0.05).

Some of our subjects showed shorter RTs in the incongruent condition than the congruent one, resulting in a negative effect of interference (ΔRT). We wanted to examine whether the directionality of interference effect is related to subjects’ levels of English proficiency or their performance in the Flanker task. We first examined whether students with a negative ΔRT showed any differences in their age of English acquisition (AoA) or test scores in the TOEFL than students with a positive (ΔRT). The results showed that there was no statistical difference in any of these measures except students with a negative ΔRT showed non-significantly higher TOEFL writing scores than students with positive ΔRTs (Supplementary Table S1).

We next assessed whether students with a negative ΔRT showed any differences in task performance. Our results showed that there was no difference in the number of correct responses made in either the congruent or the incongruent condition between students with a negative ΔRT versus positive ΔRT (for congruent condition: H_*O*_ = 1.55, df = 1, *p* = 0.22; for incongruent condition: H_*O*_ = 1.55, df = 1, *p* = 0.21). We also did not find a significant difference in the number of incorrect responses made in either the congruent or the incongruent condition (for congruent condition: H_*O*_ = 0.27, df = 1, *p* = 0.61; for incongruent condition: H_*O*_ = 0.03, df = 1, *p* = 0.85).

For RTs, we found that students with a negative ΔRT showed non-significantly higher RTs in the congruent condition than the students with a positive ΔRT (H_*O*_ = 4.56, df = 1, *p* > 0.05; Supplementary Table S2), but did not show any differences in the RTs in the incongruent condition (H_*O*_ = 1.36, df = 1, *p* > 0.05). Taken together, these findings demonstrate that students with a negative ΔRT versus positive ΔRT did not differ from each other in terms of their AoA. Their levels of English proficiency, that were assessed by the TOEFL test taken prior to the study, also did not differ at a significant *p* level of 0.05.

### Individual Differences in the Effect of Interference (ΔRTs) Were Significantly Correlated With FA Values in White Matter Voxels Connecting the VTA and the Left Caudate Nucleus

**FIGURE 2:**
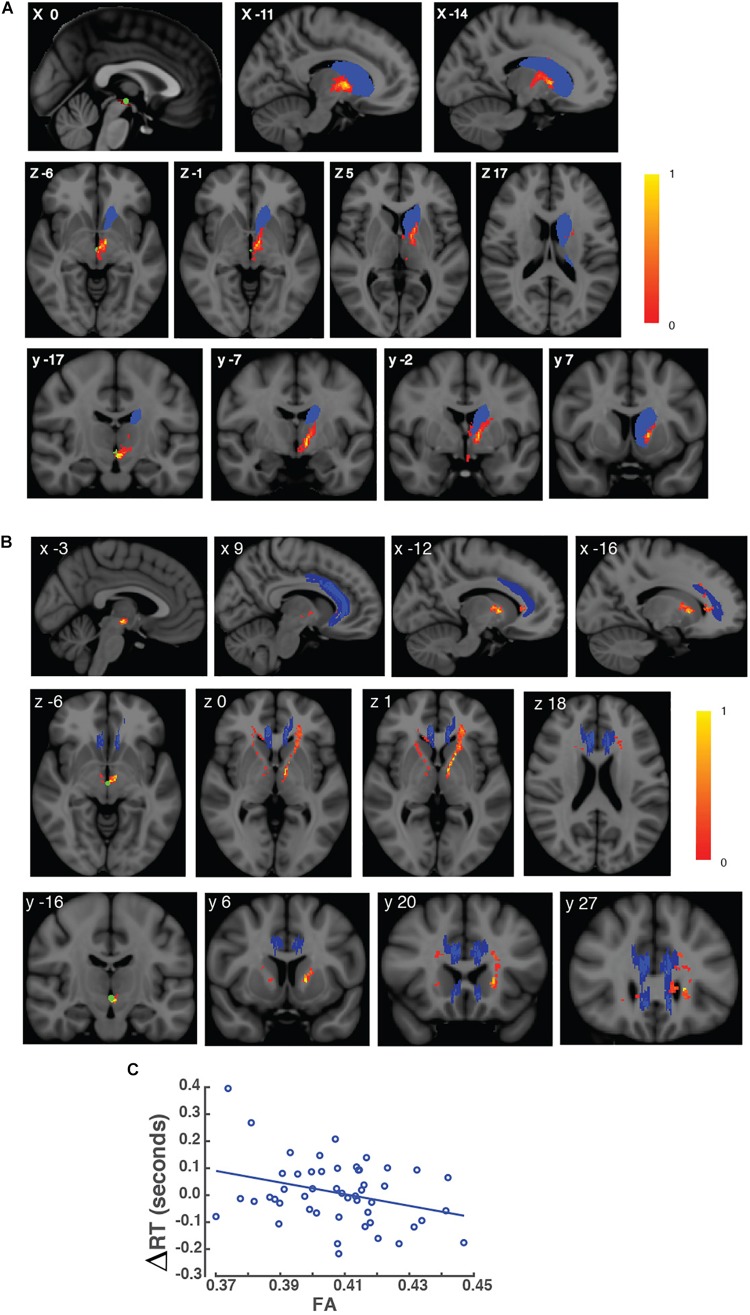
Variability in FA values in brain voxels within VTA connections. **(A)** Brain images of the VTA connections to the left caudate nucleus are shown in MNI space. Brain images are shown in a sagittal (top), horizontal view (middle), and coronal view (bottom) views. The left caudate nucleus is shown in blue, and the seed mask containing the VTA is shown in green. VTA connections to the left caudate are shown in red. The scale bar represents the values of FA in white matter voxels within the VTA connections to the left caudate nucleus. Corresponding MNI coordinates are shown in the upper left corner of each image. **(B)** Brain images of VTA connections to the ACC are shown in MNI space. Brain images are shown in a sagittal (top), horizontal view (middle), and coronal view (bottom) views. The ACC is shown in blue, and the seed mask containing the VTA is shown in green. VTA connections to the ACC are shown in red. The scale bar represents the values of FA in white matter voxels within the VTA connection to the ACC. Corresponding MNI coordinates are shown in the upper left corner of each image. **(C)** Scatter plot of the effect of interference, indexed as students’ ΔRTs, as a function of the mean FA value in voxels within the VTA connection to the left caudate nucleus from each student. *X*-axis represents the FA, and *Y*-axis represents the effect of interference (ΔRTs).

We next performed probabilistic tractography to investigate the locations of VTA connections with the caudate nucleus and the ACC. We set a seed mask in the midbrain that encompassed the VTA. FA values of voxels within these connections were used to correlate with students’ ΔRTs. We observed distinct fiber connections between the VTA and respective brain regions (Figures 2A,B). We observed an average of 18,778 (SEM = 1,988) voxels within the VTA connections to the ACC, 12,715 (SEM = 529) voxels within the VTA connections to the left caudate nucleus and 7,222 (SEM = 524) voxels within the VTA connections to the right caudate nucleus (Supplementary Figure S2). This observation is consistent with previous findings that show midbrain connections with the subcortical and cortical brain regions in non-human primates (Lynd-Balta and Haber, 1994; Heilbronner and Haber, 2014).

Our tractography-behavior analysis showed that the strength of VTA connections with the left caudate nucleus was correlated with students’ ΔRTs while controlling for age. Students with higher FA values had lower ΔRTs than students with lower FA values (partial *r* = –0.325, *p* = 0.045; corrected for multiple comparisons; Figure 2C). However, we did not find FA values in the VTA connections with the right caudate nucleus or with the ACC to be correlated with students’ ΔRTs (*p* > 0.05).

Because we observed the opposite direction in students’ ΔRTs (positive versus negative), we wanted to study whether the observed relationship between FA values in the brain voxels in the VTA connections to the left caudate nucleus and students’ ΔRTs differed between students with a negative versus positive ΔRT. Our results showed that students with either a negative or a positive ΔRT showed a significant relationship between the FA and ΔRT after controlling for age (for students with a negative ΔRT: partial *r* = –0.5383, *p* = 0.015; for students with a positive ΔRT: partial *r* = –0.4886, *p* = 0.045). We did not find FA values in brain voxels within the VTA connections to the right caudate nucleus or to the ACC to be significantly correlated with ΔRT in either students with a positive or a negative ΔRT (*p* > 0.05). Since TOEFL writing scores were non-significantly higher in students with a negative ΔRT, we entered the AoA and the TOEFL writing scores in the regression model to ensure that the significant relationship between the FA and ΔRTs was not affected by these two factors. Our multivariate regression analyses showed that FA remained significant for both groups (Supplementary Tables S3, S4). Interestingly, TOEFL writing scores also significantly predicted the ΔRTs in students with a negative ΔRT. These results confirm that FA values in brain voxels within the VTA connections to the left caudate nucleus were significantly correlated with conflict monitoring. Students’ second language skills may be related to their performance in the Flanker task. These findings support our hypothesis that students with higher FA values, indicating higher connectivity strength, were better at monitoring and reporting conflicts in arrow direction.

In order to confirm the observed relationship was not merely due to motor function, we chose the corticospinal tract as a control region to study whether the FA values in that tract correlated with students’ ΔRTs. As expected, we did not see a significant relationship between the FA values in brain voxels within the corticospinal tract and students’ ΔRTs (*p* > 0.05), suggesting that the strength of VTA connections with the left caudate nucleus was specific to students’ conflict monitoring.

## Discussion

Neural activity in the VTA is thought to be important for attention control and learning. Two brain regions that VTA neurons project to, the caudate nucleus and the ACC, showed activations when subjects monitor and report conflicts in the tasks. In this study, we sought to determine the role of VTA connectivity strength to these brain regions in conflict monitoring. We acquired DTI data from 50 young second language learners that we have previously reported on and characterized the locations and strength of these connections. We evaluated the strength of these connections by using FA and correlated it with subjects’ performance in the Flanker task. We found conflict monitoring performance to be with correlated with FA values in the VTA connections with the left caudate nucleus, but not with the right caudate nucleus, nor with the ACC. These findings suggest that the strength of VTA connections with the left caudate nucleus is associated with conflict monitoring.

Monitoring potential conflicts and adjusting behavioral performance is a crucial cognitive ability. It involves the cognitive registration of a stimulus (Posner and Petersen, 1990). Early behavioral studies have provided evidence to show that this cognitive function helps promote skill learning, including second language learning (Kellogg, 1980; Eich, 1984; Allport, 1988; Curran and Keele, 1993; Schmidt, 1995). More recent studies further demonstrated that bilingual young adults showed better conflict monitoring than matched monolingual individuals (Costa et al., 2008; Hernández et al., 2010; Luk et al., 2010; Abutalebi et al., 2012). Using the Flanker task, these studies have shown that bilingual subjects required less time than matched monolingual individuals to report the direction of a targeted arrow, suggesting an enhancement in conflict monitoring in the bilingual group. The Flanker task has been one of most commonly used tasks to assess conflict monitoring across life span (i.e., Bunge et al., 2002a; Costa et al., 2008; Treit et al., 2014; Ong et al., 2017).

There is evidence that brain dopamine levels can affect Flanker performance. Badgaiyan and Wack (2011) used positron emission tomography scans to demonstrate that there was a significant increase in dopamine release in the left caudate nucleus when subjects faced conflicting arrows in the Flanker task (Badgaiyan and Wack, 2011). Other studies that systemically administered dopamine receptor blocks to healthy volunteers also showed that reduced dopamine signaling impeded subjects’ response in the Flanker task (Zirnheld et al., 2004; Rabella et al., 2016). In monkeys, midbrain dopaminergic neurons showed excitations when monkeys were searching visual stimuli (de Lafuente and Romo, 2011), or locating a targeted image among distractors (Matsumoto and Takada, 2013). In the present study, we built upon the literature on dopamine in humans and monkeys and used the probabilistic tractography analyses to show for the first time that the strength of VTA connections to the left caudate nucleus was related to how human subjects monitored and reported conflicts in visual stimuli. Our findings corroborate previous observations of the effects of dopamine treatments on task performance in the Flanker task. We found that subjects with less connectivity strength showed poor task performance compared to subjects with greater connectivity strength of VTA connections to the left caudate nucleus. Our findings and other studies in humans and monkeys collectively suggest that dopamine levels in the left caudate nucleus are important for conflict monitoring. This signal may be broadcast from neural activity in dopaminergic neurons in the VTA.

Previous studies using DTI have demonstrated that structural properties of cortico-cortical or cortico-subcortical connections are important for various cognitive functions (Klingberg et al., 2000; Johansen-Berg et al., 2007; Floel et al., 2009; Keller and Just, 2009; Blumenfeld-Katzir et al., 2011; Sagi et al., 2012; Qi et al., 2015; Mamiya et al., 2016, 2018). In those studies, individuals with better inhibitory control or enhanced learned skills show higher FA values in those connections. One aspect of FA reflects myelination related tissue properties of white-matter structures in the brain (for a review, see Zatorre et al., 2012). It is thought that individuals with higher FA may have higher myelination, thus facilitating information flow across brain regions (Fields, 2008). We found higher FA to be associated with shorter RTs in the presence of conflicts, reflecting better conflict monitoring. These findings are consistent with many previous reports of positive correlations between FA and cognitive skills (Klingberg et al., 2000; Johansen-Berg et al., 2007; Floel et al., 2009; Keller and Just, 2009; Blumenfeld-Katzir et al., 2011; Sagi et al., 2012; Qi et al., 2015; Mamiya et al., 2016, 2018).

The findings of the present study are also consistent with previous reports of distinct roles for the ventral versus dorsal striatum in the cortico-basal ganglia-midbrain circuitry (O’Doherty et al., 2004; Fischer et al., 2017; Klein et al., 2017; Peters and Crone, 2017). These studies have suggested that the roles of the ventral and dorsal striatum in human learning are dissociable. While the ventral striatum is generally associated with reward learning, the dorsal striatum is important for monitoring and evaluating conflicts. The findings from the present study show that the strength of VTA connections with the left caudate nucleus is related to subjects’ conflict monitoring, providing evidence to show that neural connections between the VTA and the left dorsal striatum are related to human cognitive function.

The lack of a correlation between conflict monitoring and FA in the VTA connection with the right caudate nucleus, or with the ACC, may help explain why some studies show the effects of dopaminergic agents on conflict monitoring while others did not (de Bruijn et al., 2004, 2006; Zirnheld et al., 2004). It is plausible that administering non-selective pharmacological agents affects all dopaminergic pathways, and thus may not have enough impact on pathways specific to conflict monitoring.

Previous studies have reported that the ACC shows activation when bilinguals perform the Flanker task (Luk et al., 2010; Abutalebi et al., 2012). In the present study, we did not find our second-language learners’ performance in the same task to be correlated with FA in the VTA connections with the ACC. One plausible explanation for this apparent discrepancy between studies may be that the ACC is related to the overall maintenance of attention, even in the absence of conflicts (Holroyd and Yeung, 2012). In line with this idea, bilingual individuals have been reported to show brain activation in the ACC in the congruent, as well as incongruent conditions (Bialystok et al., 2005). The findings from the present study are likely more relevant to conflict monitoring in second-language learners than studies that simply demonstrate brain activation during both conditions of the Flanker task, which more likely reflects attention control in general.

One limitation of the current study is that we cannot conclude that the propagated neural connections are originating from the VTA. Probabilistic tractography does not infer the directionality of neural connections between two brain sites (Craddock et al., 2013). There is evidence that striatum output neurons can project directly to the VTA (Swanson, 1982; Watabe-Uchida et al., 2012). Therefore, neural connections between the VTA and the caudate nucleus observed in our study may contain neural pathways of the VTA projecting neurons, as well as the striatal output neurons. Future studies combining probabilistic tractography analysis and dopamine binding assays may be able to verify that individual differences in dopamine binding in the dorsal striatum are related to conflict monitoring. A second limitation is that we studied only adult second-language learners. Future studies should extend these methods to other populations.

We found RTs recorded in the incongruent, but not congruent, condition to be significantly correlated with the effect of interference on students’ task performance. This finding is consistent with an earlier report using bilingual young adults who showed enhanced interference effects only in the incongruent condition (Wu and Thierry, 2013). The study participants in the present study, as well as those in study of Wu and Thierry (2013), were young adult bilingual speakers. Neither study included individuals without second language experience (e.g., monolingual individuals). Future work is needed to determine whether the observed relationship between conflict monitoring and VTA connectivity reported here is specific to young adult second language learners or can be broadly applied to other populations.

Alert status has also been shown to affect conflict monitoring (Weinbach and Henik, 2014). Mindfulness training or specialized training in flying supersonic aircraft that foster alert status are shown to improve cognitive performance (Anderson et al., 2007; Roberts et al., 2010). Conversely, mental fatigue reduces alert status, resulting in poor cognitive performance (Faber et al., 2012). Subjects with a negative RT in the present study may have had elevated alert status during the task. Future studies may be interested in assessing how alert status and conflict monitoring interact to give rise to variations in RT in the Flanker task.

In summary, this is the first report linking VTA connectivity strength to conflict monitoring performance in humans. Our combined tractography-behavior analysis provides new evidence to show that individuals who are better at detecting and reporting conflicts also have better connectivity strength between the VTA and the left caudate nucleus. These results support the role of the VTA and left caudate nucleus in conflict monitoring. Future work may help elucidate whether these findings may also help explain variations in executive function more generally.

## Data Availability Statement

The datasets generated for this study are available on request to the corresponding author.

## Ethics Statement

The studies involving human participants were reviewed and approved by the University of Washington, Human Study Section. The patients/participants provided their written informed consent to participate in this study.

## Author Contributions

PM designed the experiments. PM and TR conducted the experiments and performed the data analysis. All authors contributed to the manuscript preparation.

## Conflict of Interest

The authors declare that the research was conducted in the absence of any commercial or financial relationships that could be construed as a potential conflict of interest.

## Footnotes

1 http://www.fmrib.ox.ac.uk/fsl/fdt/

## Supplementary Material

FIGURE S1 The distribution of the number of slices showing motion artifacts in students’ DTI images.

FIGURE S2 The average VTA connections to the left caudate nucleus. Corresponding MNI coordinates are shown in the upper left corner of each image. The caudate nucleus is shown in yellow. The VTA connections to the left caudate nucleus are shown in blue. The MNI coordinates of the mean voxel are –7(*x*), –5(*y*), and –2(*z*).

TABLE S1 Descriptive statistics of behavioral measures.

TABLE S2 Descriptive statistics of flanker responses.

TABLE S3 Statistical results for subjects with a negative conflict effect.

TABLE S4 Statistical results for subjects with a positive conflict effect.
