# Supplementary figures and images for: Strength of Ventral Tegmental Area Connections With Left Caudate Nucleus Is Related to Conflict Monitoring
Source: Front Psychol. 2020 Jan 9;10:2869. doi: 10.3389/fpsyg.2019.02869 (PMC6962310; doi:10.3389/fpsyg.2019.02869)

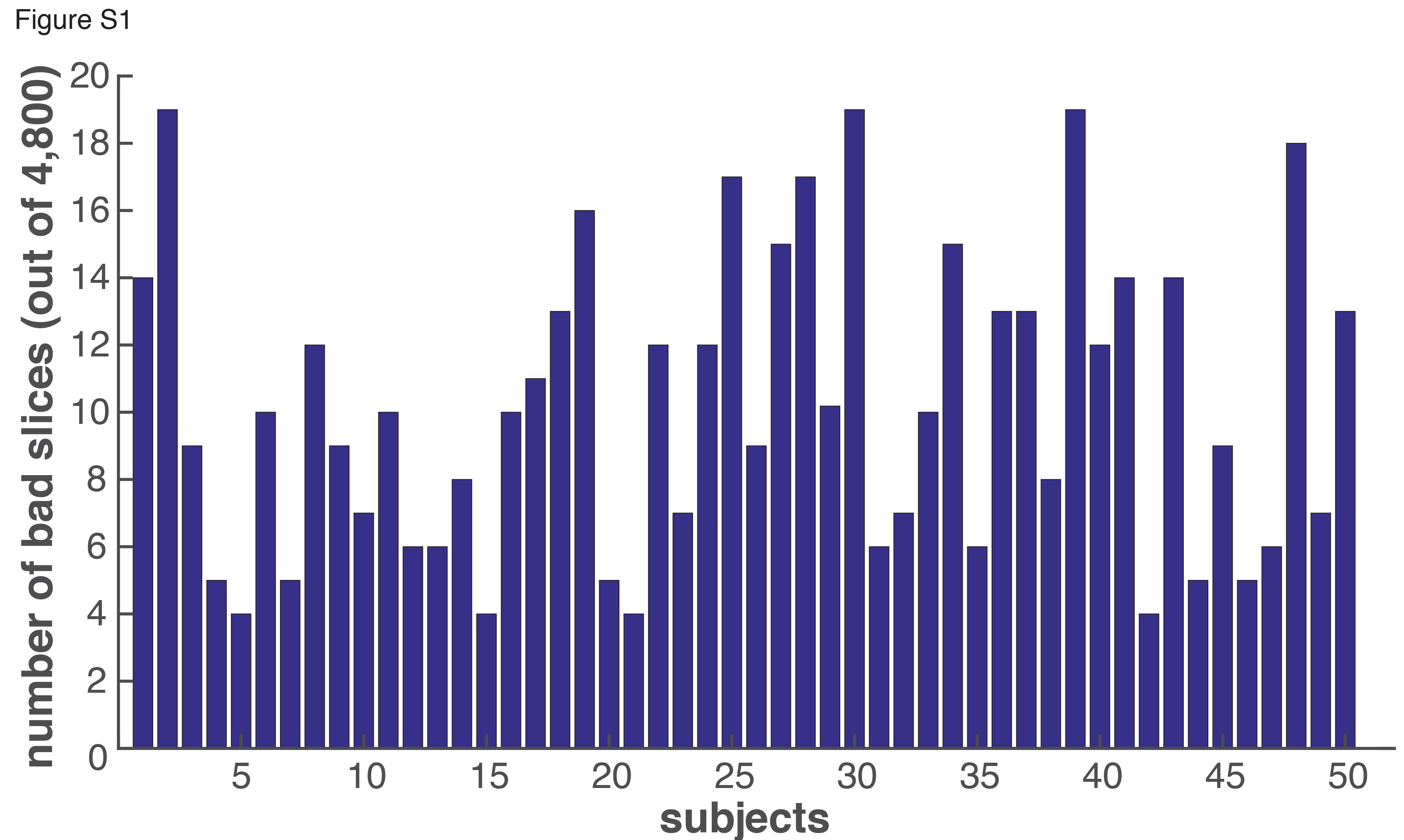

Supplement: FIGURE S1 — The distribution of the number of slices showing motion artifacts in students’ DTI images. [file Image_1.JPEG]

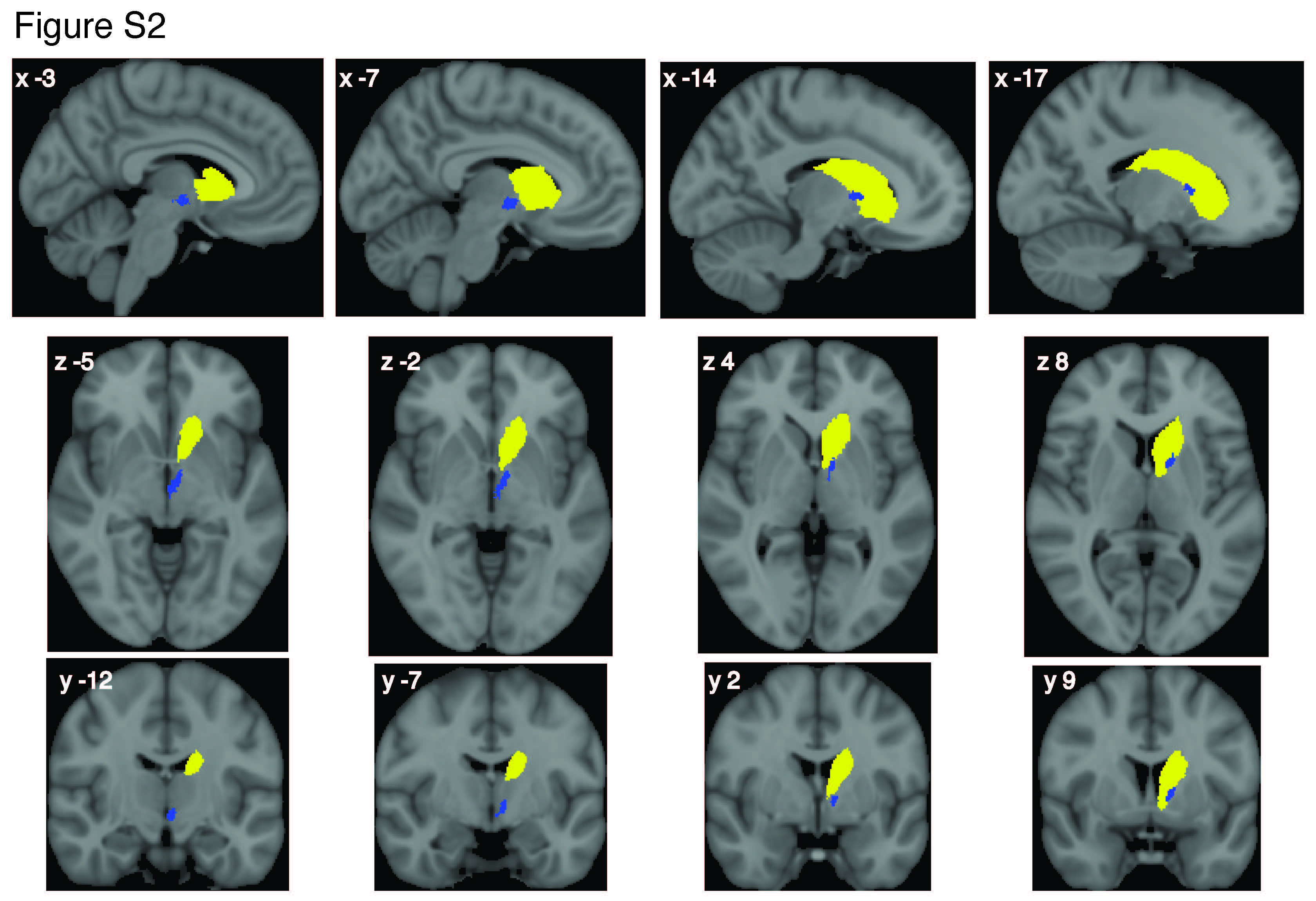

Supplement: FIGURE S2 — The average VTA connections to the left caudate nucleus. Corresponding MNI coordinates are shown in the upper left corner of each image. The caudate nucleus is shown in yellow. The VTA connections to the left caudate nucleus are shown in blue. The MNI coordinates of the mean voxel are –7(x), –5(y), and –2(z). [file Image_2.JPEG]
