# Supplementary material for: Strength of Ventral Tegmental Area Connections With Left Caudate Nucleus Is Related to Conflict Monitoring
Source: Front Psychol. 2020 Jan 9;10:2869. doi: 10.3389/fpsyg.2019.02869 (PMC6962310; doi:10.3389/fpsyg.2019.02869)
Supplement: TABLE S1 — Descriptive statistics of behavioral measures. [file Table_1.docx]

Supplementary Table S1. AoA and TOEFL scores.

|  | **Age of Acquisition (AoA)** | **TOEFL Reading** | **TOEFL Listening** | **TOEFL Writing** | **TOEFL Speaking** |
| --- | --- | --- | --- | --- | --- |
| Mean (SEM) – negative ΔRT | 8.5 (0.5) | 26.27(0.65) | 25.88(0.67) | 25.69(0.35) | 22.25(0.34) |
| Mean (SEM) – positive ΔRT | 8.4 (0.4) | 27.52(0.35) | 25.79(0.50) | 24.13(0.53) | 21.85(0.47) |
| χ2-square (df=1) | 0.0035 | 0.5017 | 0.3326 | 4.93 | 0.1492 |
| Corrected *p* values | 1 | 1 | 1 | 0.1 | 1 |

AoA: mean age of acquiring English as a second language; TOEFL: Test of English as Foreign Language; Mean(SEM) – negative ΔRT: represents the mean and the standard error of students with a negative ΔRT. Mean(SEM)-positive ΔRT: represents the mean and the standard error of students with a positive ΔRT. χ2-square and p values were derived from Kruskal-Wallis test.
