# Supplementary material for: Strength of Ventral Tegmental Area Connections With Left Caudate Nucleus Is Related to Conflict Monitoring
Source: Front Psychol. 2020 Jan 9;10:2869. doi: 10.3389/fpsyg.2019.02869 (PMC6962310; doi:10.3389/fpsyg.2019.02869)
Supplement: TABLE S2 — Descriptive statistics of flanker responses. [file Table_2.docx]

Supplementary Table S2 – Flanker responses.

|  | **Congruent condition** | | | **Incongruent condition** | | |
| --- | --- | --- | --- | --- | --- | --- |
|  | Number of correct responses | Number of incorrect responses | RTs | Number of correct responses | Number of incorrect responses | RTs |
| Mean(SEM) – negative ΔRT | 49.7(0.56) | 1.4(0.32) | 0.97(0.04) | 50.4(0.56) | 1.1(0.26) | 0.89(0.034) |
| Mean(SEM) – positive ΔRT | 50.70(0.59) | 1.5(0.34) | 0.86(0.04) | 49.3(0.59) | 1.3(0.30) | 0.96(0.048) |
| χ2-square (df=1) | 1.55 | 0.27 | 4.56 | 1.55 | 0.03 | 1.36 |
| Corrected *p* values | 0.44 | 1 | 0.065 | 0.42 | 1 | 0.488 |

Mean(SEM) - negative ΔRT: represents the mean and the standard error of students with a negative ΔRT. Mean(SEM) - positive ΔRT: represents the mean and the standard error of students with a positive ΔRT. RTs: reaction times in seconds.
