# Supplementary material for: Strength of Ventral Tegmental Area Connections With Left Caudate Nucleus Is Related to Conflict Monitoring
Source: Front Psychol. 2020 Jan 9;10:2869. doi: 10.3389/fpsyg.2019.02869 (PMC6962310; doi:10.3389/fpsyg.2019.02869)
Supplement: TABLE S3 — Statistical results for subjects with a negative conflict effect. [file Table_3.docx]

Supplementary Table S3 – Statistical results for subjects with a negative conflict effect

| A regression model with two factors | | | A regression model with four factors | | |
| --- | --- | --- | --- | --- | --- |
| R-square=0.29, *p*=0.019 | | | R-square = 0.45, *p*=0.01 | | |
|  | **F** | **P values** |  | ***F*** | **P values** |
| **Age** | 3.10 | 0.09 | **Age** | 1.69 | 0.207 |
| **FA** | 9.39 | 0.006***** | **FA** | 8.55 | 0.008***** |
|  | | | **TOEFL writing** | 5.94 | 0.024***** |
|  |  |  | **AoA** | 0.06 | 0.817 |

AoA: Age of acquiring English as a second language; Age: Students’ ages at the time of the study; FA: FA values in the brain voxels within the VTA connections to the left caudate nucleus; TOEFL writing: Scores from the TOEFL writing test.
