# Supplementary material for: Strength of Ventral Tegmental Area Connections With Left Caudate Nucleus Is Related to Conflict Monitoring
Source: Front Psychol. 2020 Jan 9;10:2869. doi: 10.3389/fpsyg.2019.02869 (PMC6962310; doi:10.3389/fpsyg.2019.02869)
Supplement: TABLE S4 — Statistical results for subjects with a positive conflict effect. [file Table_4.docx]

Supplementary Table S4 – Statistical results for subjects with a positive conflict effect

| A regression model with two factors | | | A regression model with four factors | | |
| --- | --- | --- | --- | --- | --- |
| R-squared=0.28, *p*=0.033 | | | R-squared = 0.41, *p*=0.03 | | |
|  | **F** | **P values** |  | ***F*** | **P values** |
| **Age** | 0.26 | 0.61 | **Age** | 0.02 | 0.892 |
| **FA** | 6.59 | 0.018***** | **FA** | 4.45 | 0.048***** |
|  | | | **TOEFL writing** | 1.52 | 0.232 |
|  |  |  | **AoA** | 3.32 | 0.084 |

AoA: Age of acquiring English as a second language; Age: Subjects’ ages at the time of study; FA: FA values in the brain voxels within the VTA connections to the left caudate nucleus; TOEFL writing: Scores from the TOEFL writing test.
